# Supplementary material for: Developing a predictive signature for two trial endpoints using the cross-validated risk scores method
Source: Biostatistics. 2021 Jun 24;24(2):327–44. doi: 10.1093/biostatistics/kxaa055 (PMC10102911; doi:10.1093/biostatistics/kxaa055)
Supplement: kxaa055_Supplementary_Data [file kxaa055_supplementary_data.pdf]

Biostatistics (2020), **0**, 0, *pp.* 1–23
doi:10.1093/biostatistics/SupMaterial

Developing a predictive signature for two trial endpoints using the cross-validated risk scores method: Supplementary Materials

SVETLANA CHERLIN*

Population Health Sciences Institute, Newcastle University, Baddiley-Clark Building, Newcastle upon Tyne, UK

svetlana.cherlin@newcastle.ac.uk

JAMES M. S. WASON

Population Health Sciences Institute, Newcastle University, Baddiley-Clark Building, Newcastle upon Tyne, UK and MRC Biostatistics Unit, Cambridge Institute of Public Health, Forvie Site, Robinson Way, Cambridge Biomedical Campus, Cambridge, UK

1. Simulation steps

1. Assign sensitivity status to each subject w. r. t. response 1 using Bernoulli distribution.
2. Assign sensitivity status to each subject w. r. t. response 2 using Bernoulli distribution.
3. Assign sensitivity status to each covariate w. r. t. response 1.
4. Assign sensitivity status to each covariate w. r. t. response 2.
5. Assign treatment arm status to each subject (equal randomisation).

*To whom correspondence should be addressed.

© The Author 2020. Published by Oxford University Press. All rights reserved. For permissions, please e-mail: journals.permissions@oup.com

6. Simulate the values for the gene expressions, x_{ij}, from the multivariate normal distribution conditional of the sensitivity status of the subjects and of the covariates, as specified in Supplementary Table 2.

7. For each subject, compute linear predictors ω_i for the responses $i = 1, 2$ using equations (2.3) and (2.4). The required parameters for computing ω_i are obtained as follows:

   7a. Set $\alpha_1^{(i)}, \dots, \alpha_{K_i}^{(i)} = 0$ and $\lambda^{(i)} = 0$ for $i = 1, 2$, where $k = 1, \dots, K_i$ are the indices of the covariates that are sensitive to response i.

   7b. Compute $\mu^{(i)}$ so that it corresponds to a 25% response rate on the control arm, i.e. $\mu^{(i)} = \log(0.25/0.75)$.

   7c. Compute $\gamma_1^{(i)}, \dots, \gamma_{K_i}^{(i)}$ so that they correspond to the desirable response rate in the sensitive group on treatment, RR_i, i.e.

$$\gamma_k^{(i)} = \frac{\log\left(\frac{RR_i}{1-RR_i}\right) - \mu^{(i)}}{K_i \theta_i}.$$

8. Compute probability of response as $p_i = \exp(\omega_i)/(1 + \exp(\omega_i))$.

9. Assign response i to each subject using Bernoulli distribution with parameter p_i.

2. Interpretation of the clusters

The method assumes four pre-defined clusters, based on the rate of the outcomes. For instance, if the outcomes are the safety and efficacy of a drug or a medical procedure, then the clusters can be defined as: (i) a set of patients predicted to benefit from the experimental treatment (more than average) in terms of safety and efficacy, (ii) a set of patients predicted to benefit from the experimental treatment (more than average) in terms of safety but not in terms of efficacy, (iii) a set of patients predicted to benefit from the experimental treatment (more than average) in terms of efficacy but not in terms of safety, (iv) a set of patients predicted to not benefit from the

experimental treatment (more than average) in terms of safety and efficacy. Identifying a cluster as being sensitive depends on a desirable outcome of the trial and can therefore be different in different trials. In our simulation study, it would make sense to consider cluster (i) as being a sensitive group. However, if the main concern of the trial is safety, a combination of clusters (i) and (ii) could be considered as a sensitive group. The method identifies a sensitive group that matches the definition of the true underlying sensitive group e.g. if the sensitive group corresponds to a cluster with high response rates on both outcomes then the inferred sensitive group would have high risk scores for both outcomes. The purpose is then to test the treatment effect within this cluster to show whether the predicted sensitive group does actually benefit in the predicted way.

In the case study, we assume that there are four underlying clusters of participants. In line with the notation in the simulation study, let us denote by cluster 1 a cluster that corresponds to participants who benefit from the treatment with respect to both outcomes i.e. those who have a low offence rate and a low rate of substance use. Similarly, cluster 4 corresponds to participants who do not benefit from the treatment with respect to both outcomes i.e. those who have a high offence rate and a high rate of substance use. Clusters 2 and 3 correspond to participants who benefit from the treatment with respect to one of the outcomes, i.e. participants in cluster 2 have a low rate of substance use but high offence rate, while cluster 3 has a low offence rate but high rate of substance use.

3. Computing sensitivity and specificity

To compute the cluster-wise sensitivity and specificity in the four cluster case, we assume that each true cluster in turn corresponds to a sensitive group. For example, sensitivity of a particular cluster is computed as the probability that patients who belong to this cluster by design are correctly identified as belonging to this cluster. Similarly, specificity of a particular cluster is

computed as the probability that patients who do not belong to this cluster by design are correctly identified as belonging to any other cluster. The matching of the identified clusters with the true underlying clusters is done using the distribution of the inferred risk scores, that is, a cluster with low inferred scores for both outcomes is cluster 1, a cluster with high inferred scores for both outcomes is cluster 4, and clusters with high/low inferred scores for the first outcome and low/high inferred scores for the second outcome are clusters 2 and 3.

4. Assignment of the risk scores to four clusters by marginal CVRS

The marginal CVRS separates the risk scores into four clusters as follows. Each one of the marginal CVRS analyses identifies two clusters, C_{ij}, where $i = 1, 2$ represents the responses and $j = 1, 2$ represents the clusters. Suppose $j = 1$ represents the cluster of patients that benefit from the treatment. Patients with low response rates for both responses (cluster 1) are represented by $C_{12} \wedge C_{22}$, patients with a high response rate for one of the responses and a low response rate for the other (clusters 2 and 3) are represented by $C_{12} \wedge C_{21}$ and $C_{11} \wedge C_{22}$, and patients with high response rates for both responses (cluster 4) are represented by $C_{11} \wedge C_{21}$, where the symbol $\wedge$ denotes an intersection.

5. Sensitivity Analysis

To assess the sensitivity of the CVRS2 method to various model misspecifications and extreme values of the parameters, the following scenarios (a) - (e) were investigated. The results are presented in Supplementary Table 6.

(a) Misspecification of the number of clusters.

To analyse the sensitivity of the method to the true underlying number of clusters, we analysed data simulated according to Scenario I (assuming $k = 2$ true underlying clusters of patients)

with a model that employs $k = 4$ clusters. The results presented in Supplementary Figure 3 show that most of the patients who are predicted to belong to clusters 1 and 2 correspond to true cluster 1, while most of the patients who are predicted to belong to clusters 3 and 4 correspond to true cluster 2. However, a visual inspection of the risk scores clearly shows two rather than four clusters.

(b) Misspecification of the structure of the subgroup.

In order to investigate the sensitivity of the method to a more complex underlying subgroup structure, we simulated data with prognostic effects. We assumed that there are ten covariates that increase the response rates independent of the treatment assignment. We take the response rates on the control group to be 25%, and the response ratse for the sensitive group on the experimental arm to be 70% (similarly to Scenario IIb). The response rate of 70% is composed of a combination of three factors: a baseline response rate (25%), prognostic effects of the covariates (22.5%), and the effect of treatment in the sensitive group (22.5%). Thus, due to prognostic effects of covariates, the response rate for the sensitive group on control is 47.5% (25% + 22.5%). In this scenario, we used sample size of 400. The data was analysed with a model that does not assume the presence of the prognostic effects. The results presented in Supplementary Figure 4 show low losses in sensitivity and specificity in comparison to Scenario IIb that does not include prognostic effects: the sensitivity is (0.773, 0.512, 0.687, 0.760) vs. (0.800, 0.544, 0.711, 0.782) for Scenario IIb, and the specificity is (0.998, 0.811, 0.932, 0.966) vs. (0.999, 0.830, 0.934, 0.970) for Scenario IIb.

(c) Low treatment effect for the subgroup on treatment.

To investigate the performance of the method when the treatment effect for the sensitive group is more modest, we simulated data in which the response rate in the sensitive group on the

experimental arm is 40%. In this scenario, the response rates on control was 25%. The results are presented in Supplementary Figure 5 (for $n = 400$) and Supplementary Figure 6 (for $n = 1000$). A reduction in sensitivity and specificity is observed in comparison to the results with a higher response rates (60%, 70% and 80% - see Scenarios IIa, IIb and IIc) for the sensitive group on the experimental arm, as expected.

(d) Main treatment effect without a sensitive subgroup.

As a variation of the null scenario, we considered two scenarios where there is no sensitive group but there is a main treatment effect that induces response rates of (i) 40% and (ii) 70% for both outcomes. The results are presented in Supplementary Figures 7 and 8. As expected, the sensitivity and specificity are around 25% and 75%, respectively. The response rates are estimated with a good precision (around 40% for (i) and 70% for (ii)) showing that there is no true underlying sensitive group. There is still power to show treatment effect in this case as the subgroup of patients allocated to a cluster will still benefit from the experimental treatment over the control.

(e) Misspecification of the underlying model.

In order to investigate the sensitivity of the method to a model misspecification, we simulated the data assuming that the true underlying models for the two outcomes are probit models, i.e. $p_i^1 = \Phi\Big(\mu^{(1)} + \gamma_1^{(1)} t_i x_{i1} + \cdots + \gamma_K^{(1)} t_i x_{iK}\Big)$; $p_i^2 = \Phi\Big(\mu^{(2)} + \gamma_1^{(2)} t_i x_{i1} + \cdots + \gamma_K^{(2)} t_i x_{iK}\Big)$, where Φ is the cumulative distribution function (CDF) of the standard normal distribution. We analysed these data by fitting logistic regression models as described in Section 2.3 of the main text. We assumed that the response rates on the treatment arm are 70%, the response rates on the control arm are 25% and the sample size is 400. Overall, the method was not sensitive to model misspecification (Supplementary Figure 9).

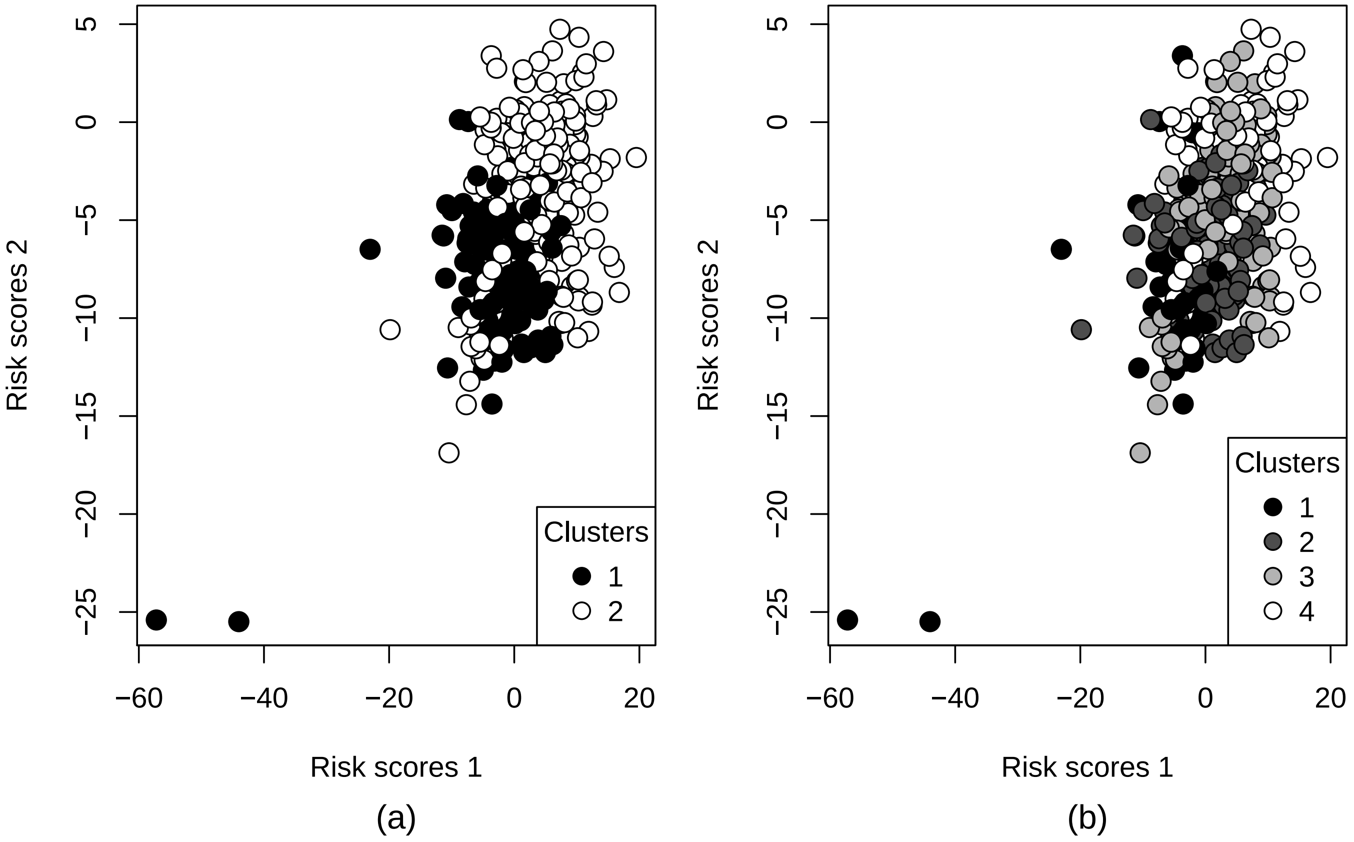

Supplementary Figure 1. The risk scores for the START data with the CVRS2 method assuming (a) two underlying clusters; (b) four underlying clusters.

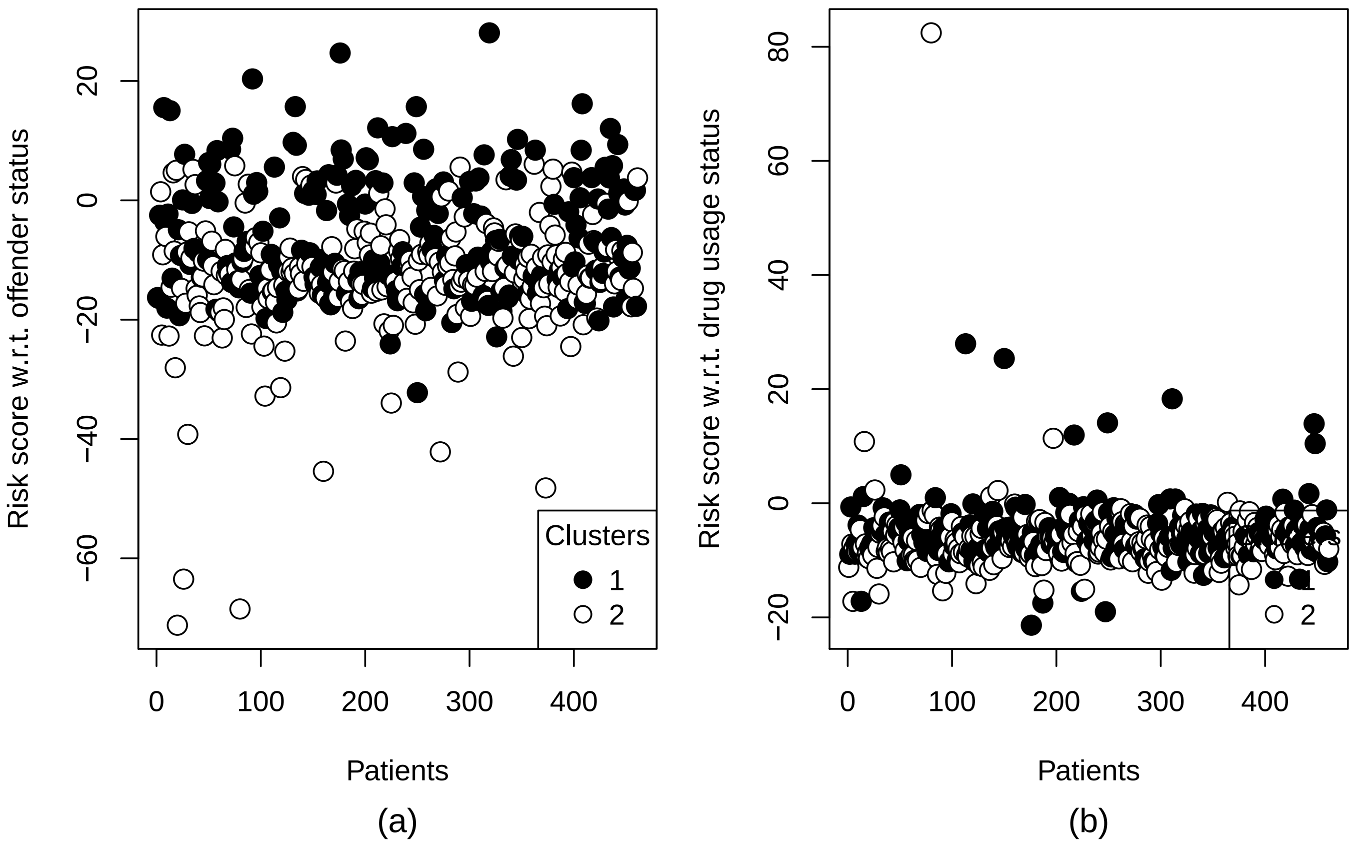

Supplementary Figure 2. The risk scores for the START data with the marginal CVRS method that was applied to the two outcome dataset (461 participants) with respect to the (a) offender status; (b) substance use status.

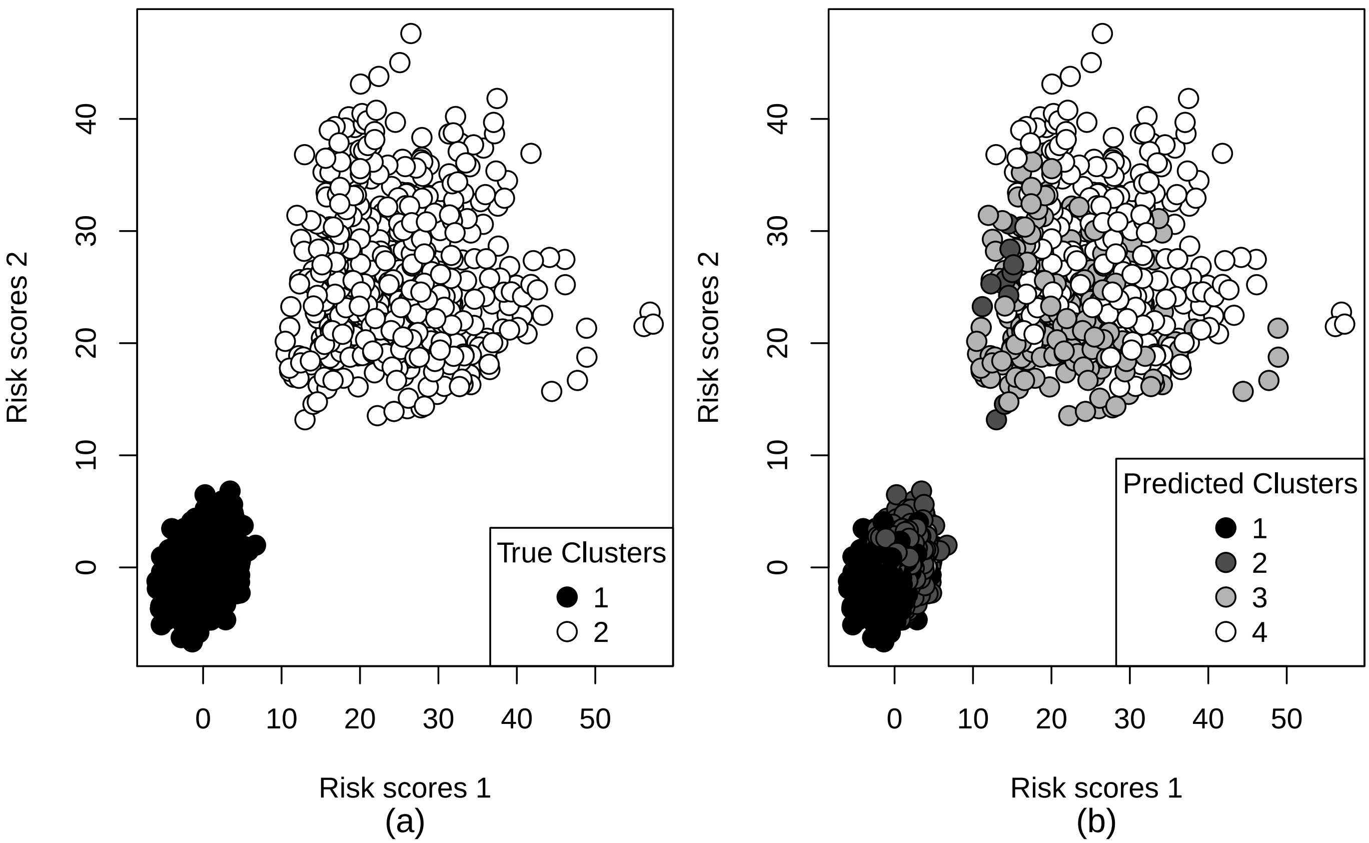

Supplementary Figure 3. The risk scores from the analysis of the data simulated according to scenario (a) “Misspecification of the number of clusters”. The data were simulated assuming there are $k = 2$ true underlying clusters and analysed with a model that employs $k = 4$ clusters.

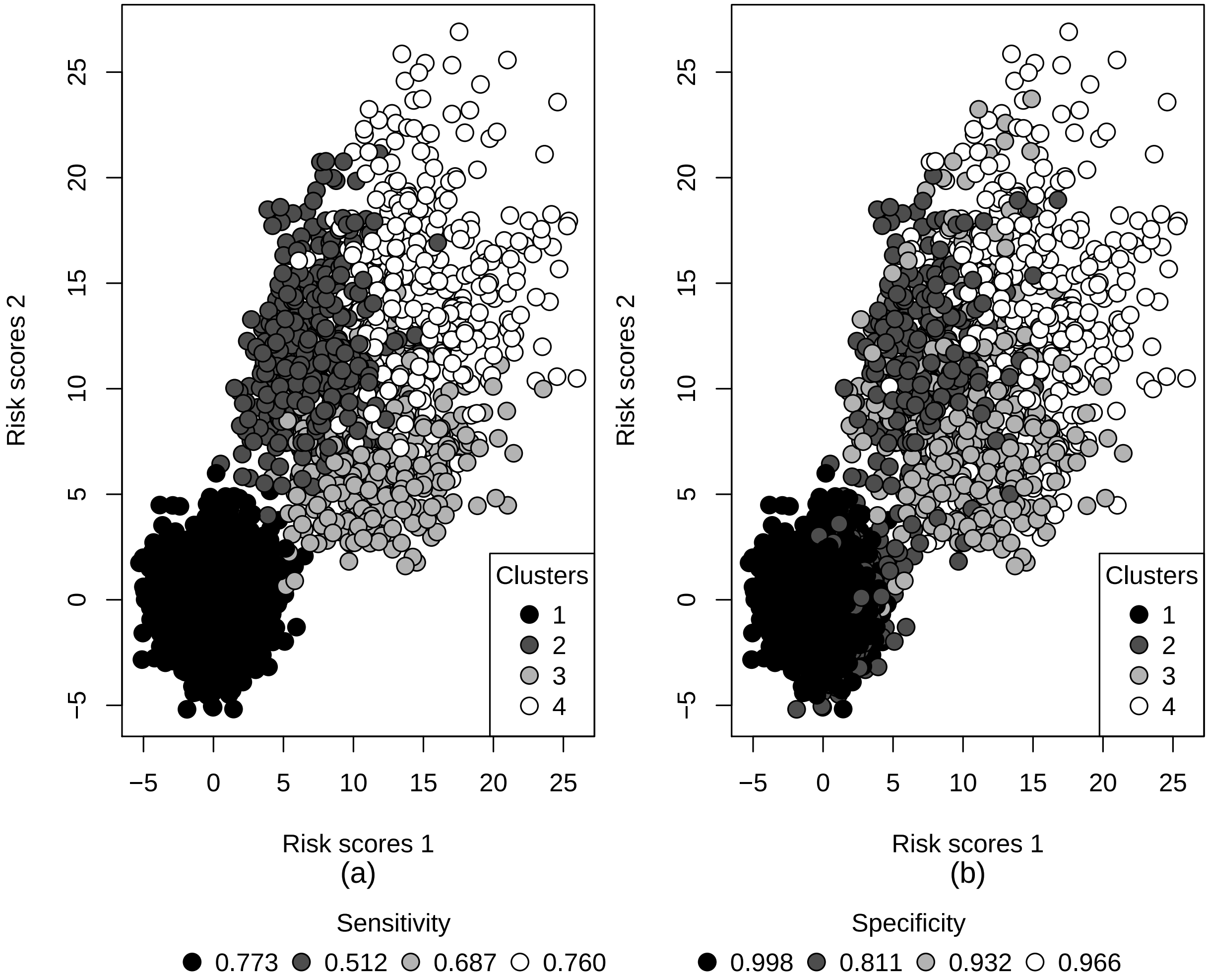

Supplementary Figure 4. The risk scores from the analysis of the data simulated according to scenario (b) "Misspecification of the structure of the subgroup". The response rates on control is 25%, the response rate for the sensitive group on treatment is 70%, the response rate for the sensitive group on control is 47.5% (due to prognostic effects of ten covariates), sample size is 400. In comparison, the sensitivity and specificity of Scenario IIb that is similar to the current scenario but for the addition of the prognostic effects, are: (0.800, 0.544, 0.711, 0.782) and (0.999, 0.830, 0.934, 0.970).

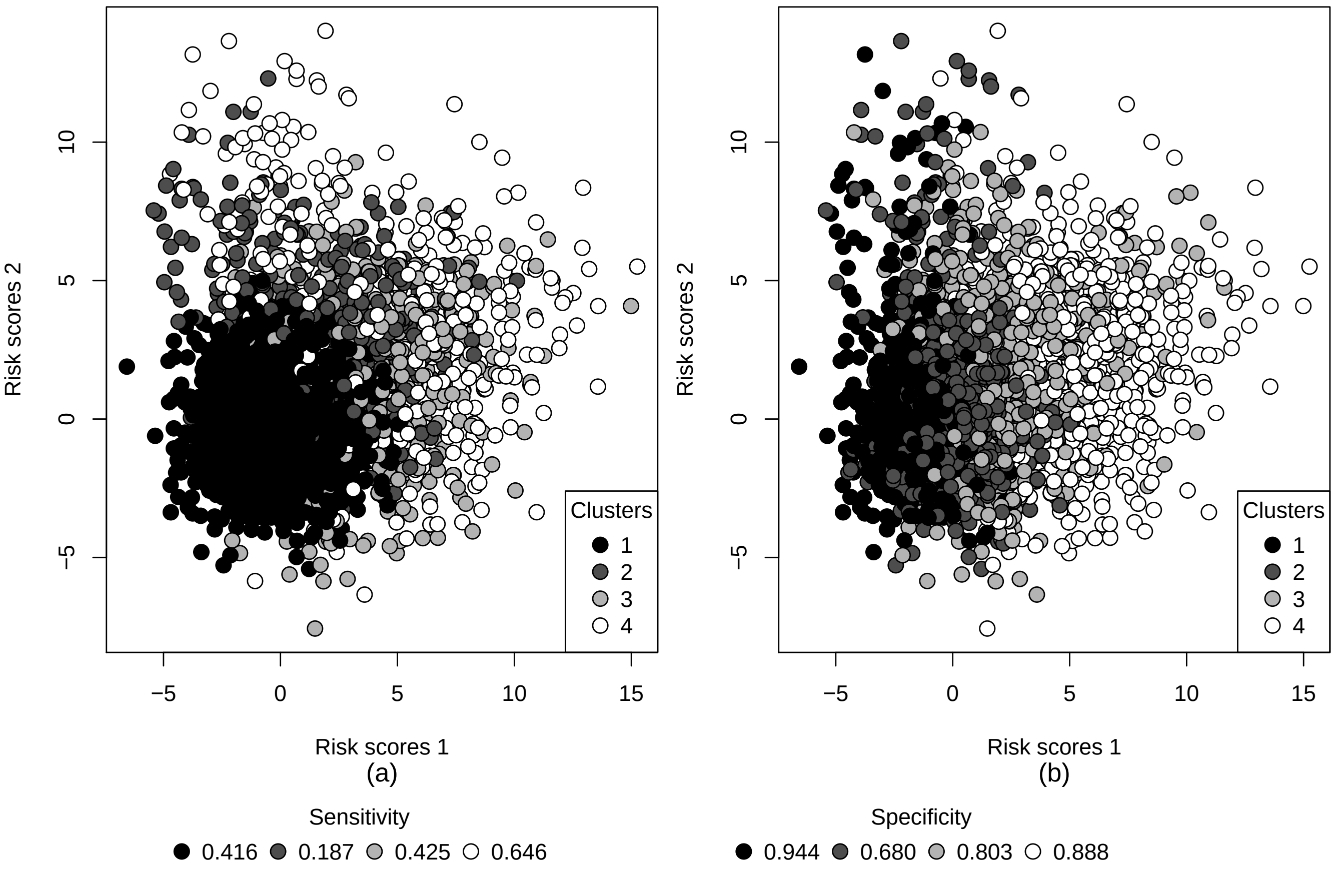

Supplementary Figure 5. The risk scores from the analysis of the data simulated according to scenario (c) “Low treatment effect for the subgroup on treatment”. The sample size is 400.

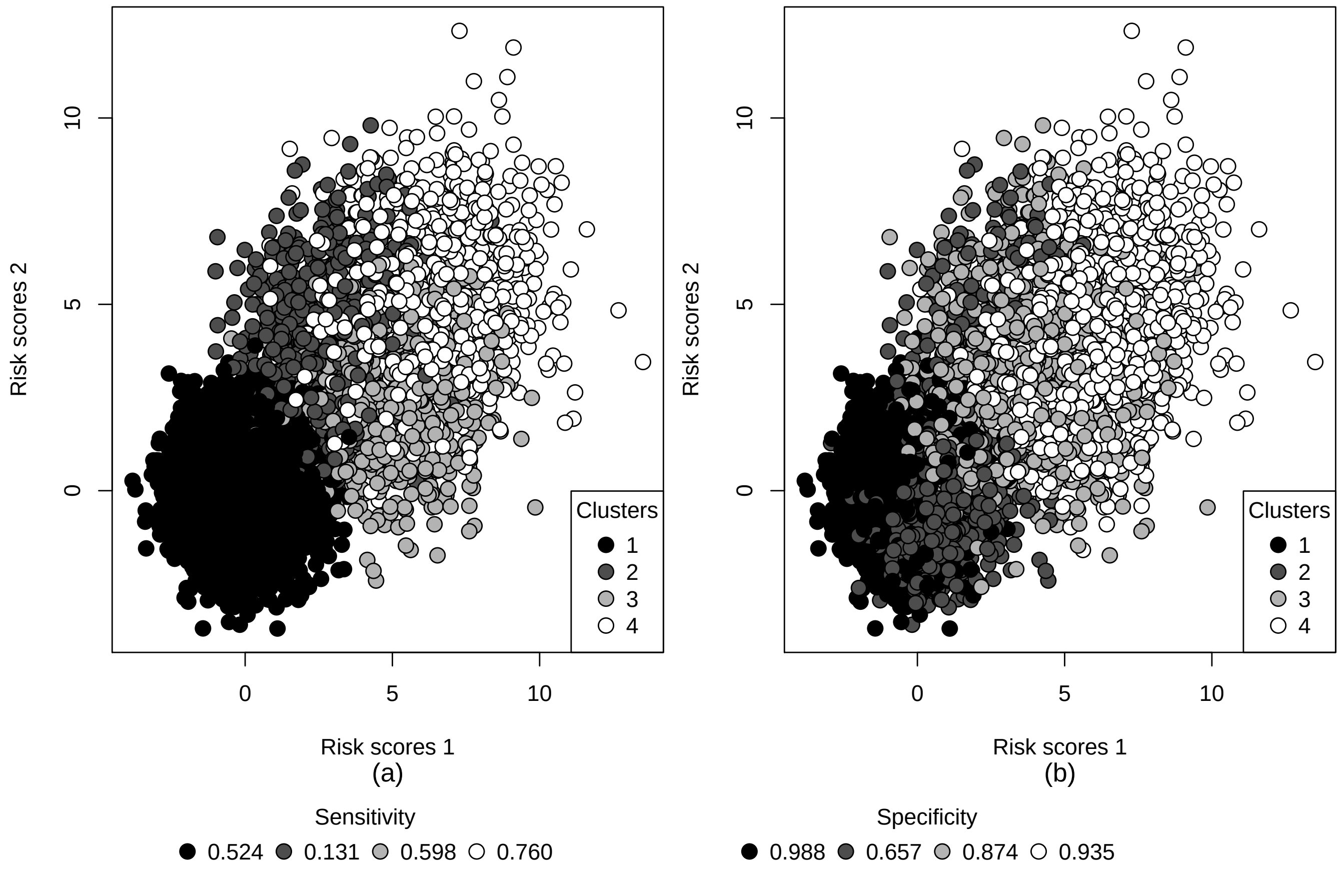

Supplementary Figure 6. The risk scores from the analysis of the data simulated according to scenario (c) "Low treatment effect for the subgroup on treatment". The sample size is 1000.

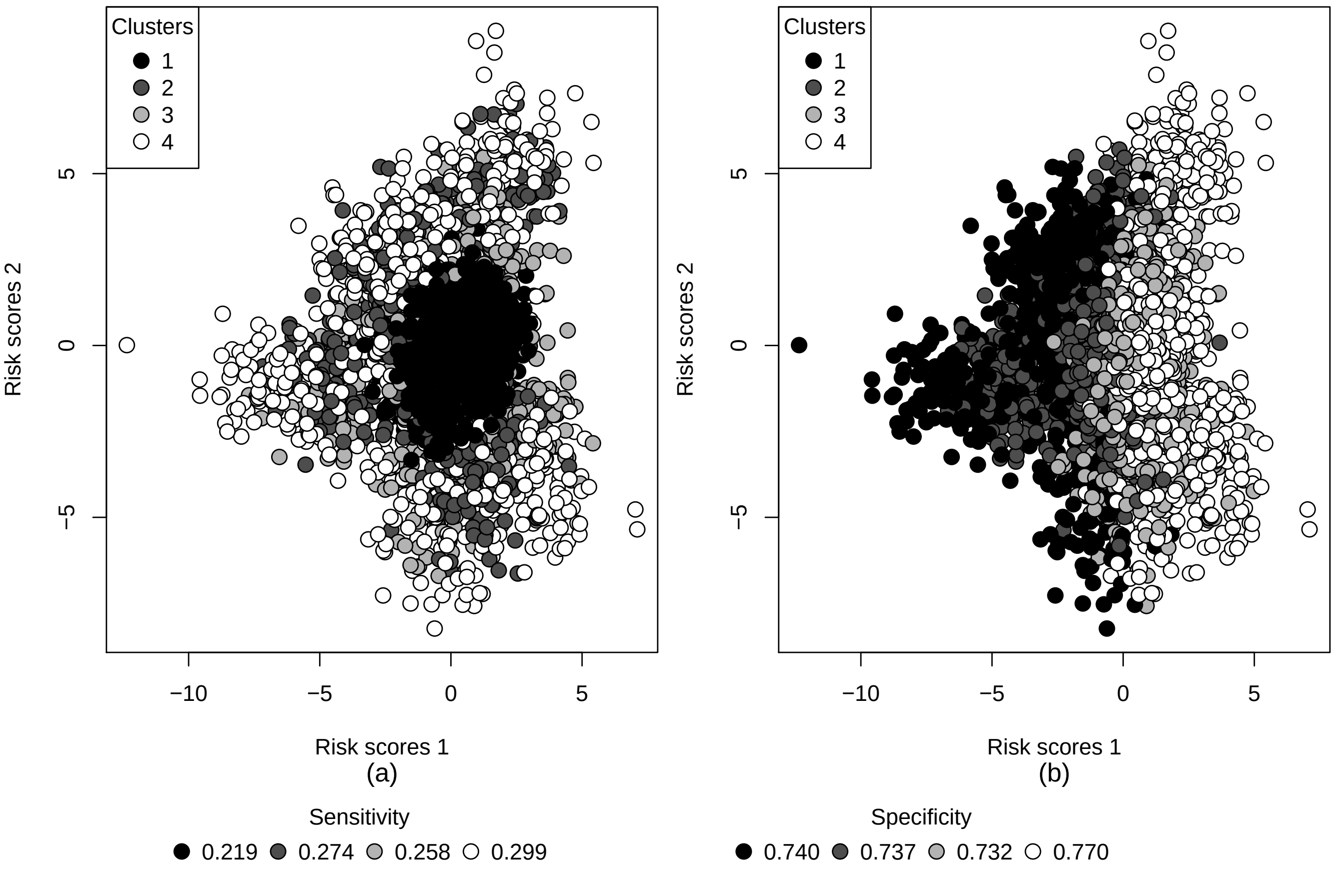

Supplementary Figure 7. The risk scores from the analysis of the data simulated according to scenario (d) "Main treatment effect without a sensitive group". The main treatment effect is 40%.

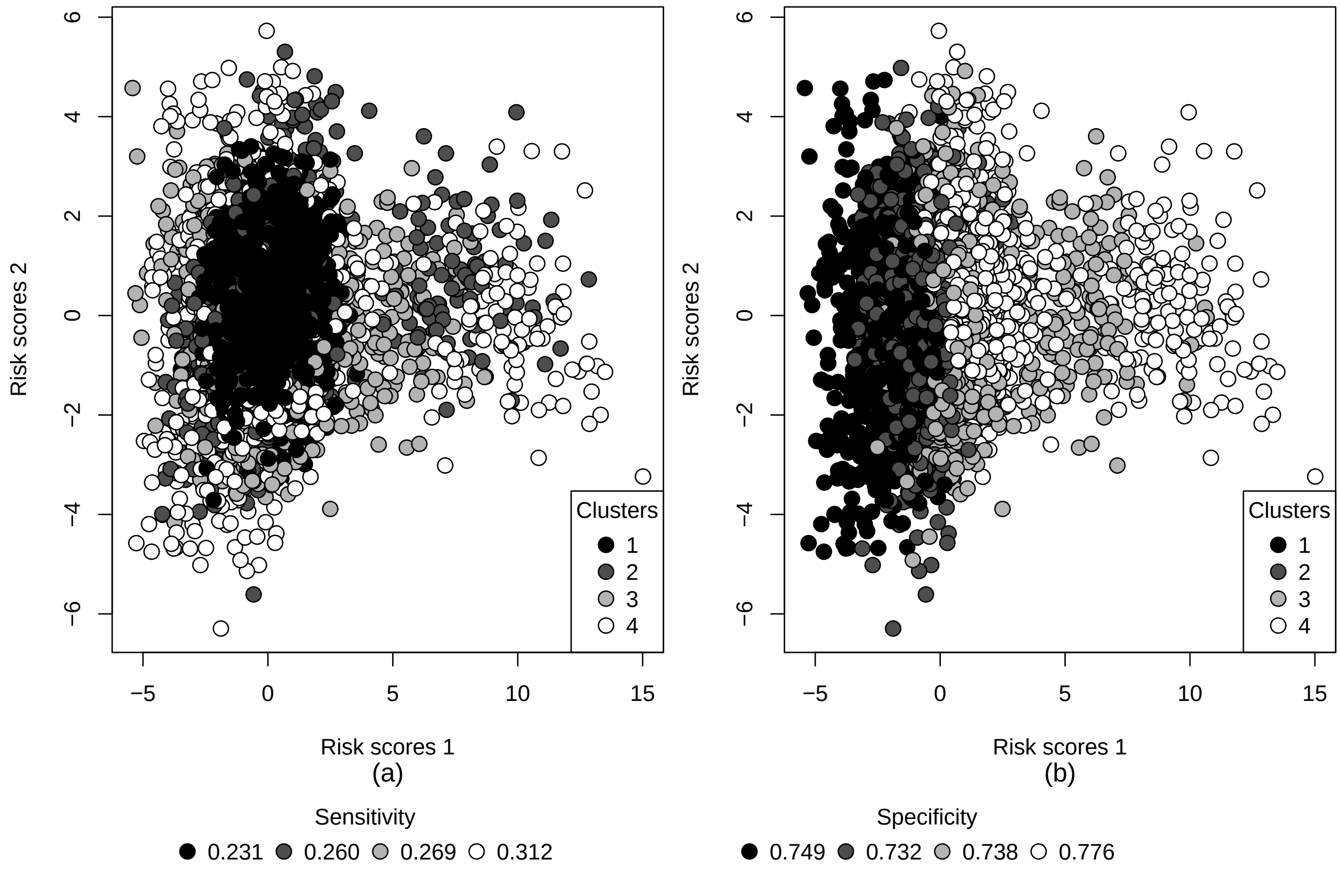

Supplementary Figure 8. The risk scores from the analysis of the data simulated according to scenario (d) "Main treatment effect without a sensitive group". The main treatment effect is 70%.

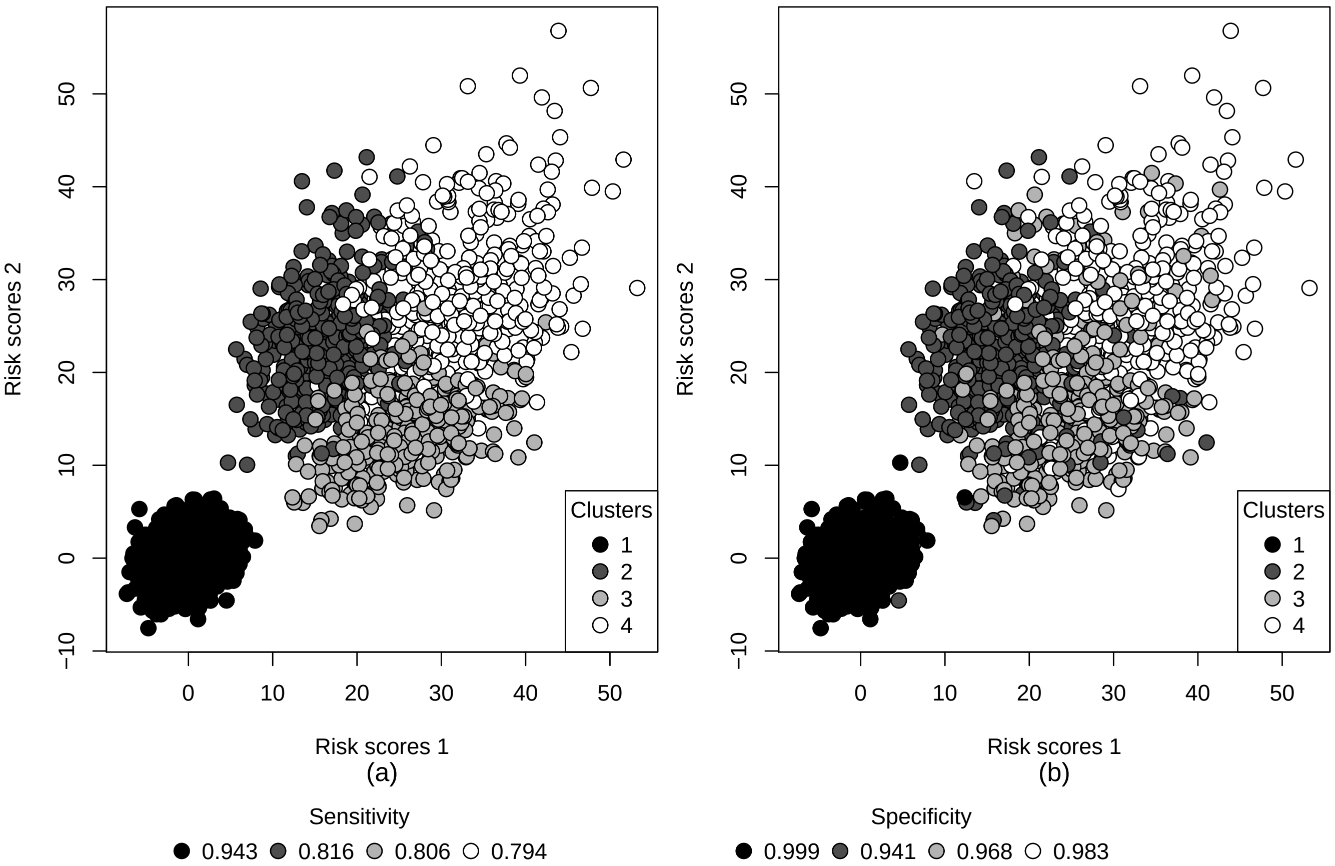

Supplementary Figure 9. The risk scores from the analysis of the data simulated according to scenario (e) “Misspecification of the underlying model”.

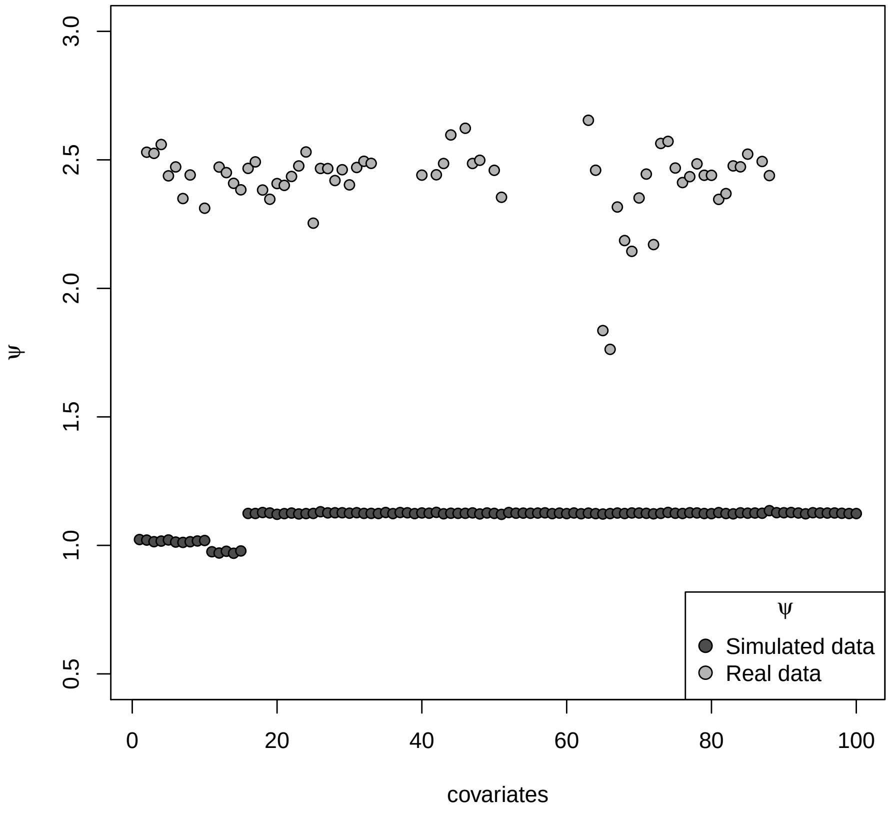

Supplementary Figure 10. A measure of association between the two binary responses, ψ, for the simulated data (Scenario IIb, average over 100 simulations, for 100 covariates) and the real data for each covariate.

Supplementary Table 1. Number of the participants in the START trial.

		Outcome 2	
		Number of substance users	Number of substance non-users
Outcome 1	Number of offenders	52 (control); 47 (treatment)	36 (control); 51 (treatment)
	Number of non-offenders	43 (control); 44 (treatment)	87 (control); 101 (treatment)

Supplementary Table 2. Parameters of the multivariate normal distribution to simulate gene expression values for different statuses of subjects/covariates. $S_i \in \{0, 1\}$ for $i = 1,\ 2$ is the sensitivity status of a subject with respect to the outcome i. $K_i \in \{0, 1\}$ for $i = 1,\ 2$ is the sensitivity status of a covariate with respect to the outcome i.

	$K_1 = 1 \wedge K_2 = 0$	$K_1 = 0 \wedge K_2 = 1$	$K_1 = 1 \wedge K_2 = 1$	$K_1 = 0 \wedge K_2 = 0$
$S_1 = 1 \wedge S_2 = 0$	$\theta_1, \sigma_1^2, \rho_1$	$\theta_2, \sigma_2^2, \rho_2$	$\theta_{12}, \sigma_{12}^2, \rho_{12}$	η, ξ^2, τ
$S_1 = 0 \wedge S_2 = 1$	$\nu_1, \zeta_1^2, \kappa_1$	$\nu_2, \zeta_2^2, \kappa_2$	$\nu_{12}, \zeta_{12}^2, \kappa_{12}$	η, ξ^2, τ
$S_1 = 1 \wedge S_2 = 1$	$\theta_1, \sigma_1^2, \rho_1$	$\theta_2, \sigma_2^2, \rho_2$	$\theta_{12}, \sigma_{12}^2, \rho_{12}$	η, ξ^2, τ
$S_1 = 0 \wedge S_2 = 0$	$\nu_1, \zeta_1^2, \kappa_1$	$\nu_2, \zeta_2^2, \kappa_2$	$\nu_{12}, \zeta_{12}^2, \kappa_{12}$	η, ξ^2, τ

Supplementary Table 3. Cluster-wise rates of responses in each arm in the START trial.

	Cluster 1		Cluster 2		Cluster 3		Cluster 4	
	control	treatment	control	treatment	control	treatment	control	treatment
Mean offender rate	0.57	0.27	0.43	0.36	0.30	0.44	0.40	0.49
Mean substance use rate	0.49	0.23	0.41	0.41	0.42	0.36	0.45	0.43

Supplementary Table 4. Covariates and coefficients for the CVRS2 method in the START trial. For each outcome, the covariates are ordered according to their coefficients.

Offender status		Substance use status	
Covarite	Coefficient	Covariate	Coefficient
YouthAdolescent	0.614	C_DAW_GenAnxT1	0.807
C_DAW_SepAnxT1	0.614	C_DAW_ADHDInattT1	0.591
RegisteredMainstreamT1	0.435	C_DAW_MajDepT1	0.525
OnsetCD	0.410	YouthAdolescent	0.504
C_DAW_ADHDCombT1	0.367	OnsetCD	0.355
C_DAW_SpePhobT1	0.340	C_DAW_SpePhobT1	0.314
YP_SDQ_CDT1	0.163	HLM_NVAP.P1	0.309
P_ALAB_CorPunT1	0.155	HLM_All.Offs.P1	0.261
YP_SDQ_HyperT1	0.145	C_DAW_ODT1	0.213
P_SDQ_HyperT1	0.132	HLM_VAP.P1	0.207
P_SDQ_EmotT1	0.129	YP_SDQ_HyperT1	0.126
YP_ALAB_PunishT1	0.116	C_DAW_CDT1	0.108
P_ALAB_MonT1	0.076	YP_SDQ_PeerRelT1	0.079
C_DAW_CDT1	0.076	YP_SDQ_CDT1	0.076
C_DAW_ODT1	0.071	YP_SDQ_EmotT1	0.076
P_SDQ_CDT1	0.071	P_SDQ_TotalImpactT1	0.068
YP_ALAB_MonitoringT1	0.050	C_DAW_ADHDCombT1	0.062
YP_SRD_Del_ExSib_VarT1	0.040	YP_SDQ_TotalImpactT1	0.056
P_CONN_ADHDTscoreT1	0.036	YP_SDQ_TotalDiffScoreT1	0.053
P_SDQ_TotalDiffScoreT1	0.029	P_SDQ_EmotT1	0.046
YP_ICU_TotalT1	0.028	P_SDQ_CDT1	0.040
IQ	0.026	P_ALAB_CorPunT1	0.031
YP_LEE_TotalT1	0.020	C_DAW_ADHDHypT1	0.028
P_ICU_TotalT1	0.019	YP_SRD_SubMis_VolT1	0.026
YP_SDQ_TotalDiffScoreT1	0.013	P_SDQ_PeerRelT1	0.025
YP_SRD_SubMis_VarT1	0.013	IQ	0.024
YP_SRD_Del_ExSib_VolT1	0.012	P_CONN_ADHDTscoreT1	0.021
YP_ABAS_TotalT1	0.012	P_ICU_TotalT1	0.020
YP_SMF_TotalT1	0.010	C_DAW_SepAnxT1	0.019
P_GHQ_TotalT1	0.004	YP_SMF_TotalT1	0.015
P_CONN_LEARLANGTscoreT1	0.002	YP_ALAB_PunishT1	0.015
Off_NOff	0.000	P_SDQ_TotalDiffScoreT1	0.012
HLM_CUST.P1	0.000	RegisteredMainstreamT1	0.012
C_DAW_SepPhobT1	0.000	P_SDQ_HyperT1	0.007
C_DAW_AgorT1	0.000	YP_LEE_TotalT1	0.006
C_DAW_OCDT1	0.000	YP_ABAS_TotalT1	0.003
C_DAW_AnxT1	0.000	Off_NOff	0.000
C_DAW_OtherDepT1	0.000	HLM_CUST.P1	0.000
C_DAW_ManiaT1	0.000	C_DAW_SepPhobT1	0.000
C_DAW_PanDisT1	0.000	C_DAW_AgorT1	0.000
C_DAW_UndiffAnxT1	0.000	C_DAW_OCDT1	0.000
C_DAW_OtherHypT1	0.000	C_DAW_AnxT1	0.000
C_DAW_OtherDistT1	0.000	C_DAW_OtherDepT1	0.000
C_DAW_SelectMutT1	0.000	C_DAW_ManiaT1	0.000

C_DAW_AttachDis_InhibT1	0.000	C_DAW_PanDisT1	0.000
C_DAW_AttachDis_DisinT1	0.000	C_DAW_UndiffAnxT1	0.000
C_DAW_AttachDis_OtherT1	0.000	C_DAW_OtherHypT1	0.000
C_DAW_PDDT1	0.000	C_DAW_OtherDistT1	0.000
C_DAW_EatDisT1	0.000	C_DAW_SelectMutT1	0.000
C_DAW_SteretypicT1	0.000	C_DAW_AttachDis_InhibT1	0.000
C_DAW_TicT1	0.000	C_DAW_AttachDis_DisinT1	0.000
C_DAW_PsychosisT1	0.000	C_DAW_AttachDis_OtherT1	0.000
C_DAW_OtherT1	0.000	C_DAW_PDDT1	0.000
YP_YouthMatScaleT1	-0.002	C_DAW_EatDisT1	0.000
YP_SRD_PeerIllSubT1	-0.006	C_DAW_SteretypicT1	0.000
RegisteredSpecialistEducT1	-0.006	C_DAW_TicT1	0.000
YP_SRD_SubMis_VolT1	-0.008	C_DAW_PsychosisT1	0.000
YP_ALAB_DiscipT1	-0.010	C_DAW_OtherT1	0.000
P_LOEB_TotalT1	-0.014	YP_SRD_Del_ExSib_VarT1	0.000
P_FACE_FlexibilityDimensionT1	-0.016	P_LOEB_TotalT1	-0.003
P_FACE_CohesionDimensionT1	-0.019	YP_SRD_SubMis_VarT1	-0.003
P_ALAB_IncDisT1	-0.026	P_CONN_LEARLANGTscoreT1	-0.003
P_SDQ_TotalImpactT1	-0.029	P_ALAB_IncDisT1	-0.004
YP_SDQ_TotalImpactT1	-0.053	P_GHQ_TotalT1	-0.004
P_ALAB_PosParentT1	-0.055	YP_SRD_Del_ExSib_VolT1	-0.007
P_FACE_FSatT1	-0.059	YP_YouthMatScaleT1	-0.008
P_FACE_FCommT1	-0.065	YP_ICU_TotalT1	-0.013
P_SDQ_ProSocT1	-0.071	P_FACE_CohesionDimensionT1	-0.015
YP_SRD_PeerDelT1	-0.073	YP_ALAB_DiscipT1	-0.019
P_SDQ_PeerRelT1	-0.084	YPEducEmpT1	-0.023
YP_SDQ_EmotT1	-0.085	P_FACE_FlexibilityDimensionT1	-0.024
YP_SDQ_PeerRelT1	-0.090	YP_ALAB_MonitoringT1	-0.034
C_DAW_PTSDT1	-0.093	P_FACE_FSatT1	-0.036
YP_ALAB_PosParentT1	-0.104	P_FACE_FCommT1	-0.057
Age	-0.108	YP_ALAB_PosParentT1	-0.067
YP_ALAB_ParInvT1	-0.113	P_ALAB_MonT1	-0.068
YP_SDQ_ProSocT1	-0.139	YP_SDQ_ProSocT1	-0.074
P_ALAB_ParInvT1	-0.146	YP_SRD_PeerIllSubT1	-0.077
HLM_OthBr.P1	-0.204	P_ALAB_ParInvT1	-0.081
C_DAW_GenAnxT1	-0.227	YP_SRD_PeerDelT1	-0.093
C_DAW_MajDepT1	-0.240	gender	-0.096
C_DAW_ADHDHypT1	-0.245	YP_ALAB_ParInvT1	-0.100
gender	-0.288	HLM_OthBr.P1	-0.117
C_DAW_ADHDInattT1	-0.448	Age	-0.123
HLM_All.Offs.P1	-0.668	P_ALAB_PosParentT1	-0.134
YPEducEmpT1	-0.720	P_SDQ_ProSocT1	-0.210
HLM_VAP.P1	-0.992	RegisteredSpecialistEducT1	-0.268
HLM_NVAP.P1	-1.549	C_DAW_PTSDT1	-0.609

Supplementary Table 5. Covariates and coefficients for the marginal CVRS method in the START trial. For each outcome, the covariates are ordered according to their coefficients.

Offender status		Substance use status	
Covariate	Coefficient	Covarite	Coefficient
C_DAW_SepPhobT1	7.461	C_DAW_PDDT1	11.345
C_DAW_OtherT1	7.192	C_DAW_PanDisT1	7.144
C_DAW_PanDisT1	7.116	HLM_CUST.P1	6.710
C_DAW_PDDT1	6.799	C_DAW_GenAnxT1	1.703
C_DAW_TicT1	4.830	C_DAW_ADHDInattT1	0.585
C_DAW_SepAnxT1	2.145	C_DAW_MajDepT1	0.507
YouthAdolescent	0.616	YouthAdolescent	0.505
HLM_CUST.P1	0.502	HLM_All.Offs.P1	0.360
RegisteredMainstreamT1	0.432	OnsetCD	0.354
C_DAW_SpePhobT1	0.430	HLM_NVAP.P1	0.315
OnsetCD	0.409	C_DAW_SpePhobT1	0.284
C_DAW_ADHDCombT1	0.367	HLM_VAP.P1	0.201
YP_SDQ_CDT1	0.162	C_DAW_ODT1	0.185
P_ALAB_CorPunT1	0.156	C_DAW_TicT1	0.130
YP_SDQ_HyperT1	0.145	C_DAW_OtherT1	0.128
P_SDQ_HyperT1	0.132	YP_SDQ_HyperT1	0.126
P_SDQ_EmotT1	0.128	C_DAW_CDT1	0.109
YP_ALAB_PunishT1	0.116	C_DAW_ADHDHypT1	0.098
C_DAW_ODT1	0.078	YP_SDQ_PeerRelT1	0.078
P_ALAB_MonT1	0.075	YP_SDQ_EmotT1	0.076
C_DAW_CDT1	0.073	YP_SDQ_CDT1	0.074
P_SDQ_CDT1	0.071	P_SDQ_TotalImpactT1	0.068
YP_ALAB_MonitoringT1	0.050	C_DAW_ADHDCombT1	0.062
YP_SRD_Del_ExSib_VarT1	0.040	YP_SDQ_TotalImpactT1	0.057
P_CONN_ADHDTscoreT1	0.036	YP_SDQ_TotalDiffScoreT1	0.052
P_SDQ_TotalDiffScoreT1	0.029	P_SDQ_EmotT1	0.047
YP_ICU_TotalT1	0.028	P_SDQ_CDT1	0.039
IQ	0.027	P_ALAB_CorPunT1	0.032
YP_LEE_TotalT1	0.020	P_SDQ_PeerRelT1	0.025
P_ICU_TotalT1	0.019	IQ	0.024
YP_SDQ_TotalDiffScoreT1	0.013	P_CONN_ADHDTscoreT1	0.021
YP_SRD_SubMis_VarT1	0.013	P_ICU_TotalT1	0.019
YP_SRD_Del_ExSib_VolT1	0.013	YP_SMF_TotalT1	0.015
YP_ABAS_TotalT1	0.012	YP_ALAB_PunishT1	0.014
YP_SMF_TotalT1	0.009	P_SDQ_TotalDiffScoreT1	0.012
P_GHQ_TotalT1	0.004	RegisteredMainstreamT1	0.010
P_CONN_LEARLANGTscoreT1	0.002	P_SDQ_HyperT1	0.008
C_DAW_AgorT1	0.000	YP_LEE_TotalT1	0.006
C_DAW_AnxT1	0.000	YP_SRD_SubMis_VolT1	0.005
C_DAW_OtherDepT1	0.000	YP_ABAS_TotalT1	0.003
C_DAW_ManiaT1	0.000	C_DAW_AgorT1	0.000
C_DAW_UndiffAnxT1	0.000	C_DAW_AnxT1	0.000
C_DAW_OtherHypT1	0.000	C_DAW_OtherDepT1	0.000
C_DAW_OtherDistT1	0.000	C_DAW_ManiaT1	0.000

C_DAW_SelectMutT1	0.000	C_DAW_UndiffAnxT1	0.000
C_DAW_AttachDis_InhibT1	0.000	C_DAW_OtherHypT1	0.000
C_DAW_AttachDis_DisinT1	0.000	C_DAW_OtherDistT1	0.000
C_DAW_AttachDis_OtherT1	0.000	C_DAW_SelectMutT1	0.000
C_DAW_SteretypicT1	0.000	C_DAW_AttachDis_InhibT1	0.000
C_DAW_PsychosisT1	0.000	C_DAW_AttachDis_DisinT1	0.000
YP_YouthMatScaleT1	-0.002	C_DAW_AttachDis_OtherT1	0.000
YP_SRD_PeerIllSubT1	-0.005	C_DAW_SteretypicT1	0.000
RegisteredSpecialistEducT1	-0.006	C_DAW_PsychosisT1	0.000
YP_SRD_SubMis_VolT1	-0.009	P_LOEB_TotalT1	-0.003
YP_ALAB_DiscipT1	-0.010	P_CONN_LEARLANGTscoreT1	-0.003
P_LOEB_TotalT1	-0.014	YP_SRD_Del_ExSib_VarT1	-0.003
P_FACE_FlexibilityDimensionT1	-0.016	P_GHQ_TotalT1	-0.004
P_FACE_CohesionDimensionT1	-0.019	P_ALAB_IncDisT1	-0.004
P_ALAB_IncDisT1	-0.027	YP_SRD_Del_ExSib_VolT1	-0.007
P_SDQ_TotalImpactT1	-0.029	YP_YouthMatScaleT1	-0.008
YP_SDQ_TotalImpactT1	-0.053	YP_ICU_TotalT1	-0.013
P_ALAB_PosParentT1	-0.055	P_FACE_CohesionDimensionT1	-0.015
P_FACE_FSatT1	-0.058	YP_ALAB_DiscipT1	-0.018
P_FACE_FCommT1	-0.064	P_FACE_FlexibilityDimensionT1	-0.024
P_SDQ_ProSocT1	-0.072	YP_ALAB_MonitoringT1	-0.033
YP_SRD_PeerDelT1	-0.073	P_FACE_FSatT1	-0.036
P_SDQ_PeerRelT1	-0.081	YP_SRD_SubMis_VarT1	-0.037
YP_SDQ_EmotT1	-0.083	P_FACE_FCommT1	-0.056
YP_SDQ_PeerRelT1	-0.088	YP_ALAB_PosParentT1	-0.067
C_DAW_PTSDT1	-0.092	P_ALAB_MonT1	-0.069
YP_ALAB_PosParentT1	-0.104	YP_SDQ_ProSocT1	-0.074
Age	-0.108	YP_SRD_PeerIllSubT1	-0.077
YP_ALAB_ParInvT1	-0.113	P_ALAB_ParInvT1	-0.082
YP_SDQ_ProSocT1	-0.138	YP_SRD_PeerDelT1	-0.094
P_ALAB_ParInvT1	-0.147	gender	-0.095
C_DAW_MajDepT1	-0.237	YP_ALAB_ParInvT1	-0.099
Off_NOff	-0.241	Age	-0.126
gender	-0.289	P_ALAB_PosParentT1	-0.134
C_DAW_ADHDInattT1	-0.440	P_SDQ_ProSocT1	-0.208
C_DAW_GenAnxT1	-0.954	RegisteredSpecialistEducT1	-0.264
C_DAW_ADHDHypT1	-0.973	HLM_OthBr.P1	-0.289
HLM_VAP.P1	-0.982	Off_NOff	-0.300
HLM_All.Offs.P1	-1.025	YPEducEmpT1	-0.475
HLM_NVAP.P1	-1.723	C_DAW_SepPhobT1	-0.530
HLM_OthBr.P1	-2.087	C_DAW_PTSDT1	-0.610
C_DAW_OCDT1	-6.210	C_DAW_SepAnxT1	-0.687
YPEducEmpT1	-12.128	C_DAW_OCDT1	-5.317
C_DAW_EatDisT1	-14.808	C_DAW_EatDisT1	-14.682

Note: The covariates that have the largest absolute values of the coefficients for the marginal CVRS, have coefficients equal to zero for the CVRS2 (for example, the C_DAW_EatDisT1 covariate which is a diagnosis of eating disorder). This is because the single-covariate regression with the *vglm* R function which is used in the CVRS2 method returns warnings "fitted values close to 0 or 1" and "some quantities such as z, residuals, SEs may be inaccurate due to convergence at a half-step" meaning that the coefficients are not reliable and therefore we set them to zero. (The warnings are most probably caused by a very small inter-subjects variability of the values of these covariates). In the marginal CVRS however, the *glm* R function computes the coefficients without a warning. For example, the C_DAW_EatDisT1 covariate has value “0” for 459 participants, value “1” for one participant and value “4” for one participant. It has the largest absolute value of the coefficient in the marginal CVRS (the coefficients are -14.808 and -14.682 with respect to the offender status and the substance use status, respectively), while for the CVRS2, the coefficients have been assigned a value of zero due to the warnings.

Supplementary Table 6. Operating characteristics for the sensitivity analysis. Scenario (a) "Misspecification of the number of clusters". Scenario (b) "Misspecification of the structure of the subgroup". Scenario (c) "Low treatment effect for the subgroup on treatment". The results correspond to sample size 400, the results in the parentheses correspond to sample size 1000. Scenario (d) "Main treatment effect without a sensitive group". The results correspond to the treatment effect of 40%, the results in the parentheses correspond to the treatment effect of 70%. Scenario (e) "Misspecification of the underlying model". The power for the trial population 0.04 level test w. r. t. response 1 and response 2, respectively is: 0.460 and 0.446 for scenario (a); 0.115 and 0.135 for scenario (b); 0.076(0.151) and 0.087(0.139) for scenario (c); 0.991(0.844) and 0.989(0.844) for scenario (d); 0.918 and 0.902 for scenario (e).

Scenario	Operating characteristics	Sensitive group corresponds to: Cluster 1	Cluster 2	Cluster 3	Cluster 4
a	Power in the sensitive group (w. r. t. resp. 1)	0.009	0.0.008	0.328	0.622
	Power in the sensitive group (w. r. t. resp. 2)	0.011	0.005	0.328	0.650
	Overall power (w. r. t. response 1)	0.464	0.464	0.613	0.786
	Overall power (w. r. t. response 2)	0.453	0.449	0.622	0.800
	Sensitivity of the group selection	0.503	0.018	-	-
	Specificity of the group selection	1.000	0.540	0.878	0.896
	Estimated rate of response 1	0.248	0.257	0.571	0.726
	Estimated rate of response 2	0.249	0.256	0.577	0.726
b	Power in the sensitive group (w. r. t. resp. 1)	0.005	0.001	0.068	0.094
	Power in the sensitive group (w. r. t. resp. 2)	0.004	0.022	0.024	0.080
	Overall power (w. r. t. response 1)	0.119	0.116	0.173	0.194
	Overall power (w. r. t. response 2)	0.138	0.154	0.155	0.202
	Sensitivity of the group selection	0.773	0.512	0.687	0.760
	Specificity of the group selection	0.998	0.811	0.932	0.966
	Estimated rate of response 1	0.251	0.271	0.567	0.676
	Estimated rate of response 2	0.251	0.399	0.439	0.611
c	Power in the sensitive group (w. r. t. resp. 1)	0.010(0.010)	0.008(0.004)	0.011(0.050)	0.055(0.177)
	Power in the sensitive group (w. r. t. resp. 2)	0.017(0.008)	0.006(0.009)	0.016(0.081)	0.031(0.113)
	Overall power (w. r. t. response 1)	0.085(0.159)	0.083(0.154)	0.086(0.192)	0.124(0.298)
	Overall power (w. r. t. response 2)	0.102(0.145)	0.092(0.146)	0.100(0.208)	0.114(0.233)
	Sensitivity of the group selection	0.416(0.524)	0.187(0.131)	0.425(0.598)	0.646(0.760)
	Specificity of the group selection	0.944(0.988)	0.680(0.657)	0.803(0.874)	0.888(0.935)
	Estimated rate of response 1	0.253(0.250)	0.263(0.253)	0.302(0.319)	0.364(0.387)
	Estimated rate of response 2	0.260(0.252)	0.263(0.258)	0.304(0.326)	0.342(0.368)
d	Power in the sensitive group (w. r. t. response 1)	0.453(0.997)	0.493(1.000)	0.510(1.000)	0.438(1.000)
	Power in the sensitive group (w. r. t. response 2)	0.416(1.000)	0.472(1.000)	0.487(1.000)	0.448(0.999)
	Overall power (w. r. t. response 1)	0.994(0.992)	0.995(1.000)	0.995(1.000)	0.994(1.000)
	Overall power (w. r. t. response 2)	0.994(1.000)	0.995(1.000)	0.995(1.000)	0.994(1.000)
	Sensitivity of the group selection	0.219(0.231)	0.275(0.260)	0.258(0.269)	0.300(0.312)
	Specificity of the group selection	0.740(0.749)	0.737(0.732)	0.732(0.738)	0.770(0.776)
	Estimated rate of response 1	0.402(0.699)	0.401(0.699)	0.403(0.700)	0.401(0.702)
	Estimated rate of response 2	0.398(0.705)	0.400(0.700)	0.399(0.702)	0.400(0.702)
e	Power in the sensitive group (w. r. t. response 1)	0.013	0.010	0.891	0.935
	Power in the sensitive group (w. r. t. response 2)	0.006	0.722	0.085	0.866
	Overall power (w. r. t. response 1)	0.920	0.920	0.989	0.992
	Overall power (w. r. t. response 2)	0.904	0.970	0.909	0.980
	Sensitivity of the group selection	0.943	0.816	0.806	0.794
	Specificity of the group selection	0.999	0.941	0.968	0.983
	Estimated rate of response 1	0.137	0.189	0.706	0.789
	Estimated rate of response 2	0.137	0.597	0.284	0.732
